# How to Manage Metallosis: A Retrospective Cohort Analysis after Revision Hip Surgery

**DOI:** 10.3390/jcm12144809

**Published:** 2023-07-21

**Authors:** Antongiulio Bruschetta, Michelangelo Palco, Domenico Fenga, Gabriele Giuca, Lukas A. Holzer, Angelo Alito, Giorgio Cacciola, Federico De Meo, Pietro Cavaliere

**Affiliations:** 1 Orthopaedic Institute of Southern Italy “Franco Scalabrino”, 98165 Messina, Italy; a.bruschetta@giomi.com (A.B.); dr.cacciola@gmail.com (G.C.); federico.demeo@gmail.com (F.D.M.); cavalierepietro@gmail.com (P.C.); 2 Department of Orthopaedic and Traumatology, Casa di Cura Caminiti, 89018 Villa San Giovanni, Italy; michelangelopalco@gmail.com; 3 Section of Orthopaedics and Traumatology, Department of Biomedical Sciences and Morphological and Functional Images, University of Messina, 98122 Messina, Italy; dfenga@icloud.com (D.F.); gabrielegiuca@outlook.it (G.G.); 4 Perth Orthopaedic and Sports Medicine Centre, Perth, WA 6005, Australia; 5 Department of Biomedical, Dental Sciences and Morphological and Functional Images, University of Messina, 98122 Messina, Italy; alitoa@unime.it

**Keywords:** metallosis, periprosthetic joint infection, tribology, corrosion, cobalt, chromium, MARS-MRI, total hip arthroplasty, metal-on-metal, pseudotumor

## Abstract

Background: Adverse local tissue reactions to metal debris are due to a metal-on-metal bearing complication caused by micromotions at modular interfaces that induce corrosion of the protective oxide layer. This process could lead to wear, fretting, and abrasion with the release of metal ions locally and systemically, which may cause adverse local reactions in nearby tissues. The aim of this study is to describe a series of patients with painful local adverse tissue reactions secondary to corrosion at the modular neck–body interface, to document the clinical presentation, diagnostic workup, and surgical findings of our research, and to search for a possible correlation between metallosis and infection. Methods: A retrospective study of patients with adverse local tissue reactions due to metal surface corrosion was performed. Blood samples were collected to identify erythrocyte sedimentation rate, C reactive protein, and procalcitonin, and a magnetic resonance imaging protocol was performed. Results: Serum cobalt and chromium levels of the 43 patients tested were significantly higher on average. However, both erythrocyte sedimentation rate and C-reactive protein were significantly elevated. Magnetic resonance imaging showed adverse reactions to metal debris with large soft tissue masses and surrounding tissue damage. Conclusions: Corrosion in hip prosthesis can lead to the release of metal ions and debris locally and systemically, resulting in local soft tissue changes. A “tumor-like” debridement can reduce this complication.

## 1. Introduction

**Figure 1:**
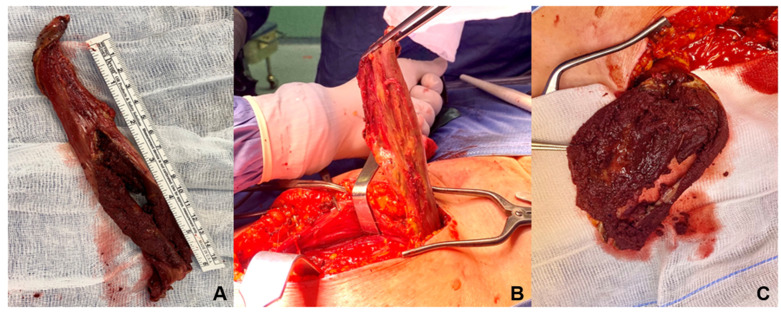
Intraoperative images of adverse local tissue reaction during revision total hip replacement surgery. Solid mass features (**A**), removal of the mass during surgery (**B**), and macroscopic appearance (**C**).

Total hip arthroplasty (THA) is one of the most effective treatments for hip joint disease, resulting in increased range of motion (ROM), pain management, and improved quality of life in most cases [1]. Over time, the development of hip joint replacement models has been the catalyst for significant improvements in both implantation methods and materials [2,3]. These include the latest bearing surfaces with larger femoral head sizes, which are designed to reduce bearing wear and the risk of dislocation while increasing the range of motion in the hip [2,4]. However, failed total hip replacements (THRs) requiring revision surgery remain an important issue and are expected to increase in the coming years [5,6]. In addition, to better restore the anatomy and motor function of the hip, modular implants are an interesting model [7]. As a result, larger diameter metal-on-metal (MoM) bearing surfaces are being widely used, particularly in young and active patients with high functional requirements [8,9]. However, these materials have a high short-term failure rate in hip surgery [10,11], with many revisions performed for adverse local tissue reaction (ALTR) [4] due to degradation and corrosion at modular prosthesis junctions [12,13,14]. Corrosion results in a higher proportion of cobalt ions being released into the joint surface and systemically [15,16]. ALTR lesions are often invasive and destructive (Figure 1) and uncertain outcomes have been reported following revision surgery [15]. To date, the literature reports over 300,000 total hip replacements per year in the USA and over 90,000 in the U.K., with rapid growth expected over the next 10 years [9,17]. Unfortunately, up to 2% of patients require revision within 18 months of surgery due to implant instability/dislocation (22.5%), mechanical loosening (19.7%), and infection (14.8%), involving both acetabular and femoral components (41.1%) [18].

**Figure 2:**
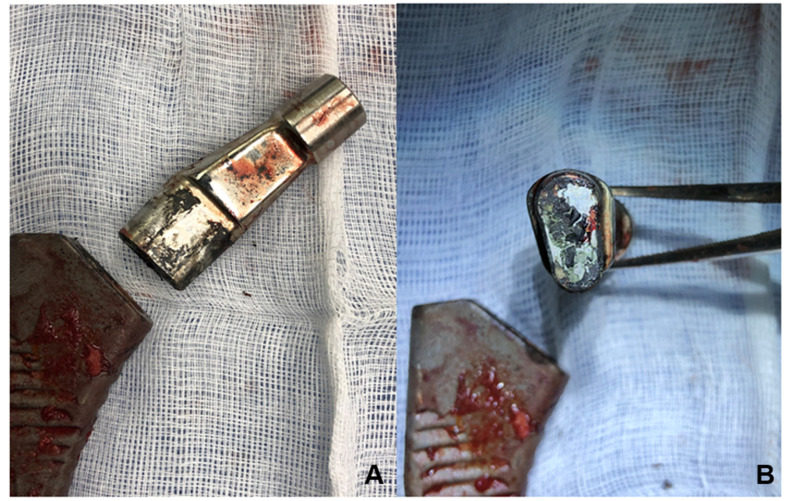
Signs of corrosion in the metal parts of a removed prosthesis at the neck–stem junction (**A**) and at the neck (**B**).

The infection rate after metal-on-metal hip resurfacing is 1.3%, which is comparable to rates after standard hip replacement [19,20,21]. The effect of metal-on-metal or metal-on-polyethylene wear debris on bacterial growth has not been reported [22]. Less common reasons for revision surgery include implant failure, osteolysis, fracture, and wear of the bearing surface [23,24]. Commonly affected prostheses are those made of titanium alloy, which can fracture causing surface cracks due to fretting corrosion [11,25]. On the other hand, a cobalt/chromium alloy prosthesis could reduce the risk of fracture due to less micromotion [10,26]. Once damage has occurred, the metal surface of the prosthesis will corrode (Figure 2) and the continuous release of metal ions could lead to ALTR [27,28].

**Figure 3:**
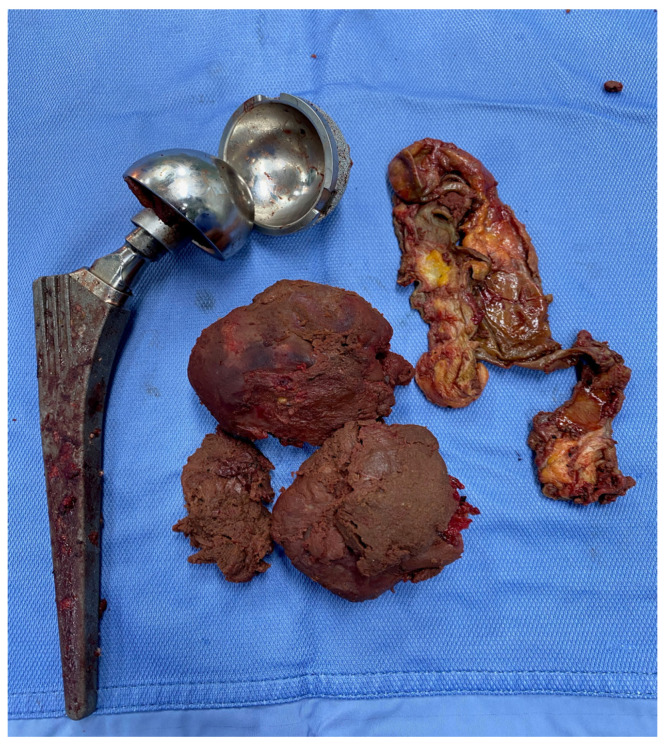
Pseudotumor and removed modular neck and metal-on-metal bearing surface prosthesis.

Such a periprosthetic soft tissue reaction can vary in size and content and is known as a pseudotumor (Figure 3) [14,29]. Several authors describe an increased rate of periprosthetic infection following metallosis [30]; similarly, a high rate of infection has been reported in patients undergoing prosthetic revision following prosthesis failure and the presence of ALRS [31].

Gill and colleagues describe their experience with a dual-modular short-stem prosthesis in which patients experienced increasing postoperative pain due to pseudotumor formation, requiring further surgery [27]. The main aim of this retrospective study is to report our experience with patients undergoing THA revision for ALTR, categorized by symptoms, and to compare after-surgery clinical outcomes on the basis of the preliminary groups.

## 2. Materials and Methods

### 2.1. Study Design and Data Collection

**Figure 4:**
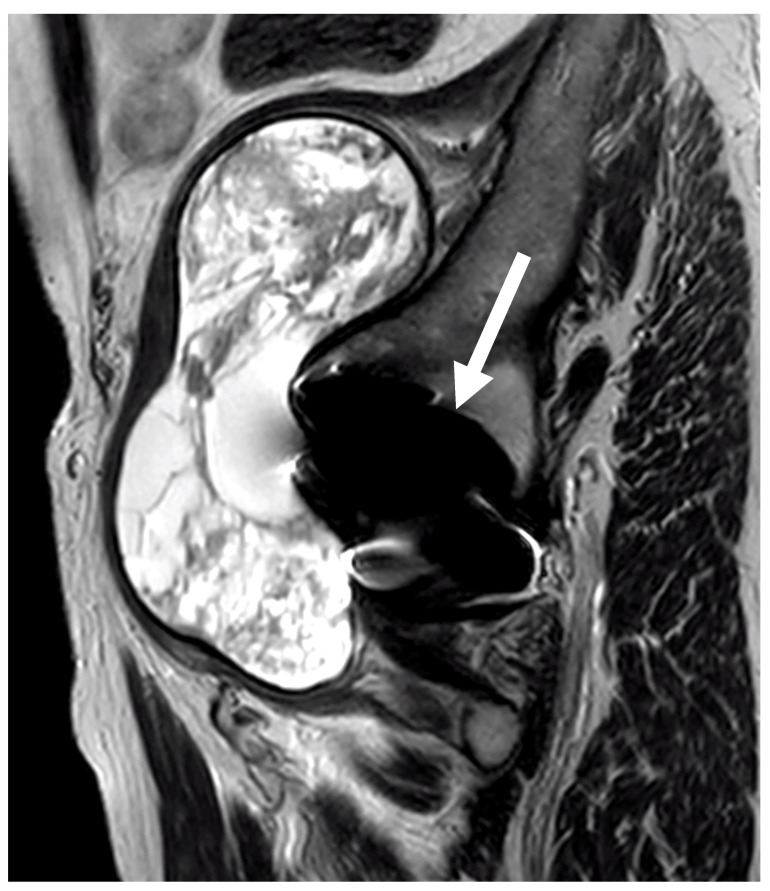
MRI image of a type III ALTR (white arrow) using MARS on the right hip.

A retrospective study of patients with ALTR due to metal surface corrosion was performed on a total of 6137 THA procedures admitted to the Orthopaedic Institute of Southern Italy “Franco Scalabrino”, Messina (Italy). After surgery, follow-up consisted of clinical examination and radiographs (anteroposterior pelvic radiographs), and radiographic findings suggestive of metallosis (evidence of bone loss or osteolytic lesions at the lesser trochanter) were referred to magnetic resonance imaging (MRI) using the metal artefact reduction sequence (MARS) protocol to measure the grade and location of the pseudotumor (Figure 4) [32]. Approximately 10% of the patients referred for MRI could not undergo the scan due to claustrophobia, various contraindications (e.g., presence of a pacemaker, stent placement incompatible with magnetic fields, or metal shrapnel in the body), or not specified “personal reasons”.

Soft tissue magnetic resonance imaging was considered pathological if the following findings were present: fluid collections, masses, or muscle changes [33]. A radiological classification system was used to classify these masses according to their degree of solidity, dividing them into three groups: Type I (thin-walled cystic masses with cyst wall < 3 mm), Type II (thick-walled cystic masses and cyst wall > 3 mm), and Type III (solid masses) [34]. Diagnosis was completed via blood tests: erythrocyte sedimentation rate (ESR) using the Westergren method (normal values 0–20 mm/h) [35], C-reactive protein (CRP) using high-sensitivity CRP tests (normal values ≥2–≤10 mg/L) [36], and cobalt (Co) (≤1.0 µg/L) and chromium (Cr) (≤1.4 µg/L) serum ion levels assessed via Quantitative Inductively Coupled Plasma–Mass Spectrometry [37]. Patients diagnosed with metallosis were divided into three groups on the basis of patient reports and clinical examination (range of motion (ROM), complete to limited), pain evaluation through numerical rating scale (NRS) [38]: slight symptoms (NRS ≤ 3, complete ROM), typical corrosion-related pain (insidious progressive groin pain, NRS ≥ 4, limited ROM) [39], and atypical pain (gluteal, thigh, or lateral pain, NRS ≥ 4, limited ROM) [40]. Blood Co/Cr levels, ESR, and CRP were compared between these groups. Patient data, implants, and surgical characteristics were obtained from medical records.

### 2.2. Characteristics of Revision Prosthesis

Prosthesis revision surgery was performed using the PROFEMUR^®^R modular stem system (Wright Medical Technology, MicroPort Orthopaedics, Arlington, TN, USA) and the MODULUS^®^R system (Lima Corporate, Villanova di San Daniele del Friuli, Italy) (Figure 3). The choice of prosthesis was related to stem geometry based on the philosophy of fitting and filling the femoral metaphyseal segment in both the mediolateral and anterior–posterior dimensions. PROFEMUR^®^R is a modular revision system with a streamlined distal stem designed to limit distal impingement and promote proximal loading, optimizing rotational instability; curved medium and long options are available to accommodate the natural femur. MODULUS^®^R achieves primary metaphyseal and proximal diaphyseal fixation through a double-tapered profile combined with a wide range of proximal bodies that provide rotational stability. At least three biopsies were taken from each patient for cultural examination, and the damaged tissue contaminated with debris was massively removed, almost like an oncological excision. The post-operative rehabilitation protocol was based on the clinical condition of the patients and generally consisted of early mobilization within 48 h, walking with the aid of a walker for seven days, and then walking with the aid of a crutch for a further three weeks.

### 2.3. Functional Evaluation

An assessment of symptoms and activity limitations using the Western Ontario and McMaster Universities (WOMAC) index [41,42] and hip disability using the Harris Hip Score (HHS) [43,44] was performed at hospital admission and 1-year follow-up. The WOMAC index is a validated self-report questionnaire widely used in lower-limb osteoarthritis; it is divided into 3 subscales assessing pain, stiffness, and disability. The total score ranges from 0 (no symptoms) to 100 (worst). The HHS is a clinician-based outcome measure used to quantify disability before and after total hip arthroplasty. The score covers different areas such as pain, function, joint deformity, and range of motion (ROM). The total score is calculated by adding the scores of the different domains, with 100 being the best score.

### 2.4. Statistical Analysis

To determine the independent predictors of corrosion-related signs and symptoms, the results were analyzed using logistic regression with ESR, CRP, chromium, and cobalt (independent variables), and then reverse stepwise regression was applied to fit the model, including imputation for missing data. Bivariate analysis was performed to describe the association between the possible predictor variables (i.e., MRI findings) and symptoms. A *p*-value lower than 0.05 was assumed to indicate a statistically significant effect. Statistical models were performed using SPSS20 software.

## 3. Results

### 3.1. Demographic Data

A total of 6137 THA procedures were performed between 2004 and 2012 using a posterolateral approach with a proximally coated, uncemented, modular neck femoral component (PROFEMUR^®^ E; Wright Medical Technology, Microport Orthopaedics, Arlington, TN; H-Max LIMA; Lima Corp., Villanova di S. Daniele del Friuli, Italy). Due to worsening pain and severe functional limitations, 395 patients (6.4%) underwent prosthesis revision after an average of 35.3 months. Among the latter, 43 patients were diagnosed with ALTR. The mean age of the 43 patients was 67.1 years (range of 30–86 years), 30 were female, 24 had a left hip and 24 a right hip, and mean BMI was 23.6 kg/m^2^ (range of 18–35 kg/m^2^). The mean interval from initial surgery to revision THA was 30.8 ± 14.5 months (range of 10–60 months). The mean follow-up after revision surgery was 40.2 ± 19.4 months (range of 20 to 75 months). Demographic data are shown in Table 1.

Different types of stems were implanted at revision considering the level of bone fixation that could be achieved. Revision femoral components were chosen among Wright and Lima corporate solutions as follows: PROFEMUR^®^R (Wright Medical Technology, MicroPort Orthopaedics, Arlington, TN, USA) (32 patients) and MODULUS^®^R system (11 patients) (Lima Corp., Villanova di S. Daniele del Friuli, Italy) using titanium alloy surface-roughened stems (Table 2). In 38 patients, the revision consisted of stem-acetabular explantation and replacement with a revision implant, in 4 cases only the stem was replaced, and in 1 case only the pseudotumor was removed. There were no intraoperative complications during the revision surgery.

### 3.2. Complications and Implant Survivorship

After revision, we recorded the following complications (12%): three dislocations treated without surgery and one stem subsidence treated with stem replacement. Moreover, three patients presented local infections associated to intense pain (NRS > 6), which were treated with antibiotic spacers and further revision.

### 3.3. Functional Evaluation

At hospital admission, the WOMAC score was 54.6 (±19.6) and 51.2 (±17.4) and the HHS was 36.5 (±5.4) and 41.4 (±3.6) for the PROFEMUR-E and H-Max groups, respectively. At one year, the PROFEMUR-R group had a WOMAC score of 11.2 (±16.4) and a HHS score of 75.4 (±5.5). In the MODULUS-R group, the WOMAC score was 10.2 (±14.8) and the HHS was 78.7 (±6.4).

### 3.4. Serum Data

In the asymptomatic group, mean ESR was 15.5 ± 9.4 mm/h (range of 2–45), mean CRP level was 2.0 ± 2.8 mg/dL (range of 0.5–16.6), mean Co level was 3.2 ± 1.9 μg/L (range of 0–7.4), and mean Cr level was 1.4 ± 1.2 μg/L (range of 0–7.0). In the typical symptomatic group, mean ESR was 14.7 ± 2.4 mm/h (range of 1–61), mean CRP was 2.1 ± 2.4 mg/dL (range of 0.1–6.1), mean Co level was 4.3 ± 2.5 μg/L (range of 0.2–11.7), and mean Cr level was 1.6 ± 1.4 μg/L (range of 0.2–8.2). In the atypical symptomatic group, the mean ESR level was 13.4 ± 0.9 mm/h (range of 1–61), the mean CRP level was 2.2 ± 0.9 mg/dL (range of 0.1–7.2), the mean Co level was 5.3 ± 3.1 μg/L (range of 0.2–14.8), and the mean Cr level was 1.6 ± 0.7 μg/L (range of 0.4–4.6). The mean Co level in all groups combined was 4.2 ± 2.6 µg/L (range of 0–13.2), with the highest Co level in a patient with atypical symptoms, normal MRI, and no other causes to explain the high blood ion levels. The mean Cr level of all groups combined was 1.4 ± 1.3 μg/L (range of 0–8.2), with the highest Cr level occurring in a patient with atypical symptoms, normal MRI, and no other causes to explain the high blood ion levels. An analysis was performed to correlate the three groups with serum levels of Co, Cr, ERS, and CRP. An overview of the blood results is given in Table 3. The reverse stepwise regression to fit the model show that no variable had a significant effect (see Table 3).

### 3.5. Microbiological Data

Three patients presented infections. At the time of debridement, the prostheses were removed and replaced with a spacer; at the same time, five deep tissue samples were taken and tested for the presence of microorganisms. Several bacteria were identified: *Klebsiella pneumoniae* in the first case, *Enterococcus faecalis* in the second, and *Staphylococcus epidermidis* in the last. Each patient received specific antimicrobial therapy before the spacer was removed in collaboration with an infectious disease specialist. Clinical features are shown in Table 4.

### 3.6. Imaging Data

The following imaging abnormalities were found. In the asymptomatic group: soft tissue changes (n.1), small fluid collections (n.2), and small soft tissue mass in the hip (n.1); in the group with typical symptoms: soft tissue changes (n.8), small fluid collections (n.4), and small soft tissue mass in the hip (n.2); in the group with atypical symptoms: fluid collections (n.5). The bivariate analysis demonstrated the presence of imaging abnormalities and/or fluid collections with or without masses in symptomatic patients compared with asymptomatic patients was statistically significant (*p* = 0.001. See Table 5). No patient had evidence of muscle damage on soft tissue imaging and the indication for revision surgery was increasingly disabling groin pain. All revisions were performed using the previous surgical technique. Regarding the presence of soft tissue masses, there was concordance between preoperative imaging and intraoperative findings in thirteen hips. There were no intra-procedural fractures during revision surgery and in thirty cases an osteotomy was required to remove the stem. Five patients had metaphyseal bone changes. The Delta TT Multihole Revision System was used in all procedures and no evidence of acetabular component damage was reported. Soft tissue imaging studies (MARS-MRI) at a minimum of two years post-revision were available for 30 patients, divided as follows. Five belonged to the asymptomatic group (17%), fifteen to the typical symptoms group (50%), and ten to the atypical pain group (33%). An overview is given in Table 5.

## 4. Discussion

### 4.1. Aim of the Study

Herein, we report our experience with the PROFEMUR^®^ modular stem system and the MODULUS-R^®^ system and their recall management.

The main aim of this study was to report our experience with patients undergoing THA revision for ALTR categorized based on symptoms. At the one-year follow-up, due to the small sample size for each group, we could not consider such group divisions, thus we analyzed the entire sample.

### 4.2. What Is Known in the Literature

Fretting and abrasion of the interfacial protective oxide layer in the modular neck–body junction and Co-Cr alloy stems could result in the release of metal ions and debris, leading to local soft tissue changes, i.e., pseudotumor [45].

Modular stem prostheses have long been used in THA [46], allowing orthopedic surgeons to separate the task of achieving stem fixation from that of setting the neck position [47,48]. The role of the modular neck stem is to provide both improved hip motion mechanics and optimal ROM regardless of the variable femoral shape [10]. Modular stems have been shown to offer advantages in restoring length and offset compared with those with exclusive head–neck joint modularity [49,50]. On the other hand, these stems also have disadvantages such as the probability of dissociation at the neck–stem junction [51,52], neck fracture [53,54], frictional wear at the joints and corrosion [55,56], resulting in the release of metal ions [57]. This phenomenon has attracted attention in the use of modular neck stems [40,45,58], as well as at the head–trunnion junction of non-modular neck stems [59]. Furthermore, where worsening and disabling pain led to revision surgery, evidence of corrosion was found in all cases, and abnormal tissue or fluid accumulation at the hip joint was identified in almost all cases. Thus, some authors have suggested a link between metallosis and infection, stating that this may be a phenomenon associated only with metal-on-metal joints, and that if metal-on-metal joints predispose glycocalyx-forming bacteria to induce necrosis to the extent that dissociation is frequent, then this would be of concern [60,61,62]. In addition, Anwar et al. found that particulate debris, of whatever composition, promote the growth of bacteria by providing a scaffold for them [22]. However, there is a possibility that patients who we previously classified as having atypical pain may also have corrosion-related symptoms.

### 4.3. Comparison between Literature and Our Data

Our data with the PROFEMUR^®^ modular stem system showed an elevated failure rate due to ALTR at the two-year follow-up (10.89%). These results differ from those reported in the literature, such as the study by Gill and colleagues, in which 3 out of 35 patients (8.6%) underwent a surgical revision for corrosion-reaction-related ALTR with neck modular stem (Eska Implants AG, Lübeck, Germany) [27]. Duwelius reported no revision cases or femoral component failures in 634 people treated with the M/L Taper hip prosthesis with Kinectiv (Zimmer, Warsaw, Indiana) [50]. Thus, a significant difference when comparing Kinectiv and PROFEMUR^®^ modular stems is the neck material composition, which is titanium alloy (Ti6Al4V) for the Kinectiv neck versus Co-Cr alloy in the PROFEMUR^®^ neck, possibly explaining the lack of corrosion-related failures in the Kinectiv modular neck. Corrosion reports resulting in ALTR in Co/Cr heads combined with non-modular stems suggest that junctions between a non-modular neck and a Co/Cr head could be a possible source of metal ions [63,64,65,66]. In our sample, all patients who reported typical groin pain after an initial clinical improvement without regression of symptoms had a progressive worsening of their clinical condition, except for one case. From an infectious point of view, the possible explanation for the low infection rate in our series is, in our opinion, due to the demolition surgery of the tissues contaminated by debris. For this reason, we suggest that care be taken during the surgical procedure to remove all contaminated material to avoid bacterial growth. No patient underwent surgery solely because of an MRI finding without a concomitant disabling clinical condition. However, ALTRs were found during revision surgery in some patients with normal imaging but significant pain symptoms. However, even in patients with few symptoms and no indication for surgery, we performed blood tests and MRI as an additional tool to guide clinical decision making. Indeed, as described by some authors, there is a decrease in Co-Cr serum ion levels after THA revision at 6 weeks after surgery, with near-normal ion levels in 98% of patients by 6 months [56,67,68].

### 4.4. Limitation of the Study and Future Perspectives

This study has the following limitations: it is a retrospective study and some data were missing. In addition, the sample size is small, leading us to consider the entire sample at the one-year follow-up, thus losing data on clinical outcomes divided per groups. Future perspectives on other retrospective studies could consider three groups in order to evaluate if belonging to the asymptomatic group correlates to a better outcome.

## 5. Conclusions

We believe that corrosion-related prosthesis failure should not be suspected only in patients who suddenly develop worsening groin pain, as even mild symptoms may be associated with ALTR. Investigation including blood inflammatory indices, serum metal ion levels, and MRI may be helpful in the diagnosis of metallosis. Revision surgery may ensure long-term clinical and functional improvement, even in the presence of infection.

## Figures and Tables

**Table 1:** Demographic data, revision mean time, and follow-up mean time.

Number of Patients	43
Age (y)	67.1 (range of 30–86)
Gender (F/M)	30/13
Side (L/R)	24/19
Revision (months)	35.3 ± 8.4
Height (cm)	157.8 ± 5.7
Weight (kg)	84.6 ± 15.4
BMI (kg/m^2^)	23.6 (range of 18–35)
Mean interval from initial THA surgery to revision (months)	30.8 ± 14.5 (range of 10–60)
Mean follow-up period after revision surgery (months)	40.2 ± 19.4 (range of 20–75)

BMI: Body Mass Index. THA: total hip arthroplasty.

**Table 2:** Functional assessment on admission and at follow-up.

WOMAC at admission for revision surgery (mean ± standard deviation) PROFEMUR-E^®^ H-max	54.6 ± 19.6 51.2 ± 17.4
HHS at admission for revision surgery (mean ± standard deviation) PROFEMUR-E^®^ H-max	36.5 ± 5.4 41.4 ± 3.6
WOMAC at one year follow-up (mean ± standard deviation) PROFEMUR-R^®^ MODULUS-R^®^	11.2 ± 16.4 10.2 ± 14.8
HHS at one year follow-up (mean ± standard deviation) PROFEMUR-R^®^ MODULUS-R^®^	75.4 ± 5.5 78.7 ± 6.4

WOMAC: Western Ontario and McMaster Universities index; HHS: Harris Hip Score.

**Table 3:** Ion levels and inflammatory markers found in patients.

	Asymptomatic	Typical Symptomatic	Atypical Symptomatic	*p*-Value
	17	16	10	
Cobalt [μg/L]	3.2 (0–7.4)	4.3 (0.2–11.7)	5.3 (0.2–14.8)	0.45
Chromium [μg/L]	1.4 (0–7.0)	1.6 (0.2–8.2)	1.6 (0.4–4.3)	0.96
ESR [mm/h]	15.5 (2–45)	14.7 (1–61)	13.4 (1–61)	0.67
CRP [mg/dL]	2.0 (0.5–16.6)	2.1 (0.1–6.1)	2.2 (0.1–7.2)	0.89

CRP: C-reactive protein; ESR: erythrocyte sedimentation rate.

**Table 4:** Clinical features of patients with infection.

Age	Sex	Time from Initial Surgery to Revision	ARMD	Microbiology
55	Female	35 months	Metallosis	*S. epidermidis*
72	Female	31 months	Metallosis	*E. faecalis*
64	Male	34 months	Metallosis	*K. pneumoniae*

ARMD: Adverse Reaction to Metal Debris.

**Table 5:** Image data findings after MARS/MRI.

		Asymptomatic	Typical Symptomatic	Atypical Symptomatic	*p*-Value
Number of patients	30	5 (17%)	15 (50%)	10 (33%)	
MARS/MRI	Muscle changes	1 (3%)	8 (26%)	5 (17%)	0.001
	Fluid accumulations	2 (6%)	4 (13%)	4 (13%)	0.001
	Mass	1 (3%)	2 (6%)	0	0.2

MARS: metal artifact reduction sequence; MRI: magnetic resonance imaging.

## Data Availability

No new data were created or analyzed in this study. Data sharing is not applicable to this article.
